# Supplementary material for: Conditional chemoconnectomics (cCCTomics) as a strategy for efficient and conditional targeting of chemical transmission
Source: eLife. 2024 Apr 30;12:RP91927. doi: 10.7554/eLife.91927 (PMC11060718; doi:10.7554/eLife.91927)
Supplement: Figure 3—source data 3. [file elife-91927-fig3-data3.zip › Figure 3 source data3 part 2.zip!/Figure 3-Source data 3-part2/1002-9-CRISPResso_Report_e53dd8m5.zip!/CRISPResso_on_1002_9/9.Alleles_frequency_table_around_sgRNA_gtatctgtccagctacgtga.pdf]

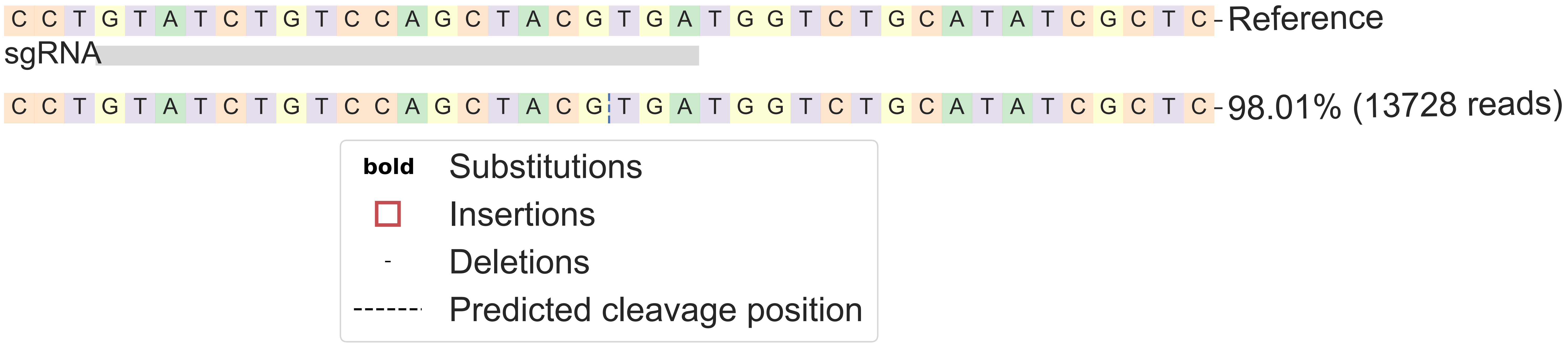
C C T G T A T C T G T C C A G C T A C G T G A T G G T C T G C A T A T C G C T C - Reference
sgRNA
C C T G T A T C T G T C C A G C T A C G T G A T G G T C T G C A T A T C G C T C - 98.01% (13728 reads)
bold Substitutions
Insertions
- Deletions
Predicted cleavage position
